# ‘Now She’s Just an Ordinary Baby’: The Birth of IVF in the British Press

**DOI:** 10.1177/0038038518757953

**Published:** 2018-02-22

**Authors:** Katharine Dow

**Affiliations:** University of Cambridge, UK

**Keywords:** 1970s, Britain, class, *Daily Mail*, emotions, IVF, media

## Abstract

The birth of Louise Brown, the first baby born through in vitro fertilisation (IVF), in England in 1978 attracted worldwide media attention. This article examines how the contemporary British news media framed this momentous event. Drawing on the example of the *Daily Mail*’s coverage, it focuses on the way in which the British press depicted Louise’s parents’ emotions, marital relationship and social class in a context of political and economic crisis and resurgent social conservatism. The British press framed the Browns as ordinary and respectable, noting their work ethic, family orientation and moral values. The article argues that the human-interest angle that the press used to represent this story created a dominant narrative in which IVF was simply a means of helping married heterosexual couples have babies and that this established a frame for subsequent depictions of IVF, as well as contributing to its rapid normalisation.

Mum and Dad were determined I would have an ordinary life. They achieved that. I have an ordinary job in my home city of Bristol and two lovely sons – conceived naturally, so I never had to go through IVF myself.

People born through IVF are just like everyone else – some are nice, some are not; some are clever, some not so clever. The first words said when I was born by the doctors who examined me were: ‘normal baby’ – that’s what I was and now I’m a normal woman. We are just normal people who needed a little help from science to get here. (Louise Brown)^1^

The birth of Louise Joy Brown, the world’s first ‘test-tube baby’, on 25 July 1978 has come to signify the pivotal moment at which the creation of humans through assisted reproductive technologies (ARTs) became a reality. The first person to be born following in vitro fertilisation (IVF), Louise Brown showed that healthy babies could be born as the result of conception outside the body. IVF has gone on to be one of the most influential technologies of the last four decades. More than a quarter of a million IVF babies have now been born in this country alone (HFEA, 2016). IVF has served as a platform for new technologies like pre-implantation genetic diagnosis and regenerative medicine and has kick-started a global fertility industry that is predicted to be worth $21.6 billion by 2020 (Moran, 2015). ARTs have brought about new possibilities for people to create both biogenetically related children and novel family formations, with new legal regulations and institutions emerging alongside them.

Louise’s birth in Oldham, a post-industrial town in north-west England, attracted sustained worldwide media coverage (Nelkin and Raymond, 1980; Van Dyck, 1995; see also Anker and Nelkin, 2004). News media have been influential in informing, reflecting and shaping public debate and opinion about ARTs (Birenbaum-Carmeli et al., 2000; Franklin, 1990; Michelle, 2007; Mulkay, 1997; Petersen, 2001; Salazar and Orobitg, 2012; Shalev and Lemish, 2012; Williams et al., 2003). In the UK, the media, despite their frequent ambivalence about reproductive technologies (Franklin, 2013; Turney, 1998), have played a significant role in the relatively rapid normalisation of IVF (Condit, 1994; Franklin, 1990, 2013; Michelle, 2007; Mulkay, 1997; Seguin, 2001), yet surprisingly little research has been done on the way they have represented IVF in the UK.

Before Louise’s birth, her parents Lesley and John Brown signed a syndication deal with Associated Newspapers, on the advice of Patrick Steptoe and Robert Edwards, the consultant obstetrician and research scientist who helped them achieve their pregnancy (Edwards and Steptoe, 1980). The *Daily Mail*, whose circulation was then 1.8 million (Greenslade, 2003: 345), consequently had exclusive rights to the story in the UK. Their coverage was generally more accurate on the specifics of the Browns’ case since they had close access to the key sources, but the story was covered across all newspapers. As unofficial biographer of the *Mail*, Adrian Addison, has recently written, it has always been a right-wing and socially conservative paper aimed at men and women readers and with a consistent editorial voice, proud to be the voice of middle England. Addison (2017: 189) describes Margaret Thatcher, who became prime minister in 1979, and David English, the *Mail*’s editor during the 1970s, as ‘firm friends and political soulmates’. Crucial to its robust moral stance is its valorisation of the institution of the family, which English articulated in May 1978 in relation to another story: ‘The *Daily Mail* is a family newspaper, which means it cares passionately for those values of family life that bring so much to ordinary people in this country’ (quoted in Addison, 2017: 193).

This article examines how complex interactions between media tropes, political debates, economic change, social attitudes and classed moralities created the dominant narrative of the birth of Louise Brown in the British press in 1978. Focusing on the coverage in the *Daily Mail*, I will analyse how Louise’s parents were represented. The Browns were a working-class couple who happened to be the first ones whose IVF pregnancy went to term following trials with hundreds of volunteers in the Oldham clinic established by Steptoe and Edwards (Elder and Johnson, 2015). They were consequently, and reluctantly, thrown into the media spotlight, but came to be seen as deserving parents of the world’s first IVF baby, largely because of their apparent ordinariness.

It is important to critically consider the way in which the media framed the event of Louise Brown’s birth since, as Carolyn Michelle (2007: 640) points out in her study of media representations of ARTs in New Zealand/Aotearoa, ‘Media […] comprise a major source of information used by individuals, interest groups and decision-makers to construct understandings of the social and policy issues raised by new scientific and medical developments, which are generally outside most people’s direct experience.’ While there was limited interest in IVF’s potentiality in the popular press before 1978, the media coverage of Louise Brown’s birth was intense, not just in the UK, but around the world. The implication that IVF was only really newsworthy once a child had been born suggests a presumption among journalists and editors that IVF was primarily about family, marriage and parenting – an expectation that is reflected in the ‘human-interest’ frame that the different newspapers adopted in representing this story. But, as Stuart Hall’s (Hall et al., 2013) work reminds us, it is important not only to understand the frame that journalists in 1978 used to represent the advent of IVF in humans, and the kinds of shared meanings that that reproduced, but also to consider what alternative frames were disregarded.

The dominant narrative in the British press during 1978 was a largely positive one with little space given to the kinds of ethical questions that would flavour the public debates of the 1980s (see Mulkay, 1997). The ethical questions raised in 1978 were more to do with good parenting, the institution of the family and the professional standing of medics and journalists than ontological questions about the status of the human embryo or concern about what novel family formations this technology might enable.^2^ The press’s highly sympathetic coverage of the Browns’ story in 1978, which was consistent across newspapers, suggests that there was a willingness to accept this ‘brave new’ technology, whatever ethical, political and social questions it came to raise. Rather than the kind of dystopian speculations about what IVF might lead to that emerged in later representations of ARTs, and which were present earlier in the century (see Haldane, 1924; Squier, 1994), this origin story was one in which science was simply assisting a natural need and bringing happiness to an ordinary, loving heterosexual marriage.

This article focuses on two interlinked aspects of the press’s contemporaneous representation of Louise Brown’s parents: first, their emotions, both in terms of their personal characteristics and the quality of their marriage; and second, their ‘ordinariness’, which is closely related to both their class position and heteronormative expectations of family life. The decision of the British press to frame this story as a human-interest one that paid close attention to the ‘ordinary’ needs and emotions of Lesley and John Brown helped establish IVF as a technology that was about providing hope to the infertile and assisting the ‘normal’ and ‘natural’ desire to have a biogenetically related child. While the *Daily Mail* might be expected to present a normative account, the story of what IVF was, what it meant and who should use it remained consistent across the press, so that a ‘common-sense’ view of IVF emerged.

The socio-historical context of the time undoubtedly played a crucial part in the establishment of this dominant narrative. The year 1978 signified a crossroads between the post-war socialism and social progressiveness of the 1960s and early 1970s and the conservatism and economic neoliberalism that accelerated under Thatcher. As well as widespread concern about the trade unions’ power that culminated in the Winter of Discontent, this was a time of debate about sexuality, gender and ‘the family’. While of course Thatcher did not enjoy unanimous support and this was a lively time for various left-wing campaigns, the dominant culture was shifting towards greater social conservatism. The 1970s had seen significant social and demographic changes in the UK. The divorce rate, numbers of single-parent families and teenage pregnancies all rose during the 1970s, causing consternation among more socially conservative commentators (Sandbrook, 2012: 402). Many newspapers voiced concern about the decline of ‘middle-class values’ and the preservation of the institution of the family, as well as fears about working-class people becoming dependent on government help, having more children than they could afford to raise and not respecting authority (Sandbrook, 2012; Todd, 2014; see also Cannell, 1990; Mulkay, 1997: 14).

In this article, I will present the data in some detail in order to establish a more nuanced historical picture, and to demonstrate the broader sociological significance of this under-researched part of the story of assisted reproduction. The data analysed here illustrate how the media both shape public understandings of new technologies and establish what kinds of questions they seem to raise. Considering the political, economic and social context of the time in which Louise Brown was born reminds us that decisions to represent IVF as being only about happy families, ‘desperate’ infertile women or scientists ‘playing god’ – whether by journalists, academics, patients or clinicians – are political ones and should be understood as such. Such claims should therefore be approached with a critical eye.

I argue that the human-interest angle that the press used to represent this story created a dominant narrative in which IVF was portrayed as simply a means of helping married heterosexual couples have babies and that this contributed to its rapid normalisation as well as establishing a frame for subsequent depictions of IVF. The press’s decision, epitomised in the *Daily Mail* coverage, to depict the birth of Louise Brown to a modest and respectable working-class couple as the origin story of IVF was a powerful means for neutralising the more potentially problematic aspects of IVF, because it created a tight association between an ‘ordinary’ family and this extraordinary technology. This helped lay the groundwork for IVF’s normalisation, as well as putting the onus on critics to counter this association in their assessments of the technology and its applications.

## Methods and Data

I have collected over 140 articles about Lesley Brown’s pregnancy and Louise’s birth from all national daily newspapers (the *Daily Express*, *Daily Mail*, *Daily Mirror*, *Daily Telegraph*, *Financial Times*, *Guardian*, *Sun* and *The Times*), published between 1 January and 31 December 1978, which I accessed in the British Library’s archives. The *Daily Star* was not included since it did not start printing until November 1978. The coverage of Lesley Brown’s pregnancy and Louise’s birth was extensive during this year, starting in April, peaking in July when Louise was born and continuing until late in the year in most cases.

In this article, which takes a qualitative approach, I will focus on the *Daily Mail*’s coverage of the story, because it was vital in the formation of the dominant narrative of the birth of Louise Brown that emerged in 1978, given the paper’s exclusive access to the primary sources. Lesley and John Brown published a memoir, *Our Miracle Called Louise*, shortly after Louise’s birth, co-written with a freelance journalist (Brown et al., 1979). This and a recent memoir by Louise Brown herself (2015a) provide helpful additional material for understanding the family at the centre of the media storm.

## Showing Your Feelings

Louise’s conception and birth was the culmination of many years of trying – for Robert Edwards and Patrick Steptoe,^3^ who had spent much of their careers working on reproductive issues, and for the Browns, who had been trying to get pregnant for 14 years (Brown et al., 1979: 13). Lesley and John, who died in 2012 and 2007 respectively, were both born and bred in Bristol. They had been together since Lesley was 16 years old. John already had two daughters from his previous marriage. After John’s first wife left the family to pursue a new relationship, one was adopted and the other, Sharon, was taken into care (Brown et al., 1979: 27). Soon after John and Lesley started co-habiting, they collected Sharon from the children’s home. She lived with them from then on and apparently thought of Lesley, who had spent part of her own childhood in a children’s home, as her mother ever after.

The Browns married after six years, once John had secured his divorce, but often let people believe they were married while they were still co-habiting, as extra-marital sex, while becoming more common, was still something of a taboo. Their relationship seems to have been a strong and loving one. As they told it, the only consistent problem, which caused both of them emotional distress and led to periods of severe depression for Lesley, was the fact that they had been unable to conceive a child together. After years of intermittent medical consultations and investigations, Lesley was referred to Patrick Steptoe, 180 miles away in Oldham, and enrolled into his and Robert Edwards’ private research programme on IVF (Brown et al., 1979). In the following sections, I will describe the affective framework that emerged for the story of the birth of Louise Brown through the *Mail*’s accounts of her parents’ emotions and marital relationship.

### Shyness and Determination

The personal characteristic most often attributed to Lesley by the press was shyness – combined, and contrasted, with her apparently extraordinary determination. The day before Louise’s birth, the *Daily Mail*’s Lynda Lee-Potter wrote an article about the Browns called ‘The baby the whole world is waiting for’. Her description distils the way in which Lesley’s personality was represented in the contemporary media:

One of the most moving aspects of this remarkable birth is that it centres on Lesley Brown. She is not an emancipated, fearless, intrepid woman of today. She’s a shy, home-loving introvert who finds great difficulty in expressing her feelings. She’s taciturn and pathologically terrified of hypodermics, but she persisted in her quest long after more articulate, seemingly confident women would have given up.

[…] Lesley seems gentle and unsure on the surface but underneath she’s implacable. Her overwhelming physical need to hold her own baby to her breast was so powerful it overcame her innate shyness, her modesty, her reticence, her terror of hypodermics.

She says: ‘When doctors tell you you’re never going to have a child of your own, you ought to believe them. They’re the clever ones. You ought to accept what they say but something inside me all the time was telling me they were wrong.’ (Lee-Potter, 1978a: 16)

Lynda Lee-Potter, who died in 2004, was the *Mail*’s star columnist and interviewer. She was one of their main journalists to work on the story once the exclusive had been secured, writing a number of profiles of the Browns. Lee-Potter was suspicious of feminism, obsessed with the class system and a champion of social mobility (*Daily Telegraph*, 2004; Maddox, 2004). She was particularly interested in the emotional appeal of stories (*Daily Telegraph*, 2004). She could also be scathing (Cozens, 2004; Leapman, 2000; Maddox, 2004), though as Adrian Addison (2017: 171) notes, she was put under pressure by editor David English to develop this acerbic style after her predecessor Jean Rook defected to the *Express*. Given this style and her particular views about class and respectability, it is notable that Lee-Potter painted an extremely positive, even romantic, picture of the Browns. Presumably there would have been editorial pressure to present the story in a way that would help the *Mail* sell as many copies as possible given their investment in the syndication deal, and the decision seems to have been made that a positive and celebratory human-interest take on the Browns’ story was the best way to achieve this.

In the above extract, Lee-Potter dealt Lesley a number of backhanded compliments which put her firmly in the camp of being the ‘shy, home-loving introvert’, the polar opposite to the ‘emancipated, fearless, intrepid woman of today’. Many other newspapers described Lesley as a housewife, and although she did give up her job around this time – presumably because her employers would not accommodate her regular visits to Oldham – she had been in employment since she left school. Lee-Potter’s opposition between ‘emancipated’ and ‘home-loving’ women, which could also be glossed as the ‘feminist’ and the ‘housewife’, would have had a particular resonance in 1978. Feminism had achieved much (though especially for middle-class, white women), especially in relation to sex and reproduction by 1978, but it was still viewed as militant and hysterical by many right-wing commentators including Lee-Potter, so her favourable representation of Lesley as firmly in the ‘housewife’ mould was placing her on one side of the fence in a context of resurgent conservatism.

‘Human-interest’ stories like this one of Lesley’s quest to become a mother play upon and reproduce binary oppositions between culture and nature, reason and emotion and male and female as well as perpetuating the privileged authority of scientific knowledge (Franklin et al., 1991; see also Williams et al., 2003). The trope of Lesley as housewife, coupled with her ‘innate shyness’ laid the ground for her to be accepted as a suitable mother for Louise. Lesley’s determination could be explained, according to Lee-Potter, by her ‘overwhelming physical need to hold her own baby to her breast’. This explanation, which perpetuates the popular idea that women’s strongest natural drive is to become a mother, along with her quiet stoicism, allowed Lesley to appear feminine despite her single-minded behaviour and the fact that she was having a baby through technological assistance. It also showed an early association between the ‘innate’ desire to have biogenetically related children and the ‘success’ of IVF, which has become an important trope in winning popular and political support for ARTs (Franklin, 2013), alongside the recurring idea that Lesley’s pregnancy provided ‘hope’ for infertile women (see also Franklin, 1990).

### ‘Go and find a proper wife’

Journalists agreed that the Browns’ marriage was a good one and so Louise’s birth was a happy ending not only in the sense that it fulfilled their – but especially, Lesley’s – desire to have a child, but also in that it seemed to ensure that, in a climate of concern about rising divorce rates, this marriage would endure. The Browns’ marriage had, in fact, been put under great strain by infertility (Brown et al., 1979), a point that was picked up by some journalists. In the *Mail*, Lesley described how she had felt after her gynaecologist had told her that she had a million-to-one chance of conceiving a baby. In a few poignant lines, she got to the heart of the devastating effects that infertility can have on a person’s identity and relationships: ‘You feel you’re not the same as ordinary wives. You don’t feel normal. You feel you’re not a real woman. I said to John, “Go and find a proper wife”’ (Lee-Potter, 1978a: 17).

Journalists portrayed Lesley as quiet, strong and determined, while, in contrast to gender stereotypes of working-class men, John was depicted as emotionally expressive, sensitive and self-sacrificing. Before Louise was born, John talked about his and Lesley’s discomfort expressing their emotions and how this had changed through the pregnancy:

‘We’ve not been people to show our feelings before this happened’, says 38-year-old railway lorry driver, John. ‘Before, if I’d said, “I love you, darling”, I’d have looked down and been embarrassed. Now I can look at [Lesley] in the eyes and tell her what she means to me.’

‘When I walk into that hospital room knowing what she’s been through, I just feel so much emotion inside it’s as though my throat will burst.’

‘We’re not ashamed of our emotion. When I go in to see Lesley I’m sweating. My heart starts beating faster. I can’t wait to get in that room, give her a big kiss and say, “Hello, my love”. To think she’s got our baby inside her is so much that it almost overwhelms me.’ (Lee-Potter, 1978a: 16)

John attributed his newfound ability to physically and verbally express his emotions to the impending birth of his daughter, but he also implied that the expression of feelings was secondary to having them – he and Lesley always loved each other deeply even if they felt embarrassed saying so before. This reticence would have chimed with the British value of emotional reserve, which is particularly associated with the middle and upper classes. John’s description of his physical sensations is also typical of romantic love, which counters the separation of sex and reproduction entailed in finally conceiving Louise, while allowing him to claim emotional attachment to both Lesley and the foetus she was carrying.

While Steptoe and Edwards were subject to some criticism over the years,^4^ the Browns themselves were never depicted negatively, despite being the centre of media attention. This had the effect of contributing to a consensus that they were suitable and deserving parents for the world’s first ‘test-tube baby’. As Sara Ahmed (2014: 4) notes, claims to and representations of emotions and emotionality are ‘dependent on relations of power, which endow “others” with meaning and value’. Describing Lesley’s happiness, joy and relief at becoming a mother at last as well as her martyr-like fortitude was a way of helping readers sympathise with this reticent woman who was very uncomfortable in the media spotlight. Focusing on Lesley’s emotions, which Lynda Lee-Potter explicitly linked with her natural instincts, reinforced her feminine and maternal qualities and thereby established an association between IVF and the heteronormative family. The particular emotions associated with Lesley and her marriage to John also fitted classed ideas of appropriate emotions. As I will discuss in the next section, the Browns were represented as a respectable and somewhat socially mobile couple and their personal qualities of shyness and modesty played an important part in this. In attributing particular emotions to them, journalists framed who the Browns were and what their story meant – and the effect in this case was precisely not to other them, but to normalise them. Journalists were sympathetic to this white, heterosexual married couple and the story that emerged was one of happiness created by the birth of their long-awaited blonde, blue-eyed daughter.

As Carolyn Michelle (2007: 641; see also Michelle, 2006; Petersen, 2001; Priest, 1994) writes, ‘news stories may actively “frame” issues in ways that legitimate particular understandings of them, while simultaneously excluding or downplaying other possible explanations, thereby narrowing the scope of public debate’. Human-interest stories are employed to make new technologies more meaningful for general audiences (Michelle, 2007). They are also perceived to sell more papers, especially by tabloids, so they are the result of economic, as well as stylistic, considerations (Van Dyck, 1995). Whether or not they take an explicit human-interest angle, media representations of IVF and ART are infused with emotions, as when journalists talk of ‘public fears’, ‘ethical concerns’ or ‘relief’ or ‘hope’ at a particular outcome.

Given the traditional gendered separation of ‘hard’ and ‘soft’ news, which is usually associated with broadsheet and tabloid newspapers respectively (Pantti, 2010), the framing of IVF’s beginnings as a human-interest story might have been criticised by broadsheet papers as ‘sensationalist’, yet in 1978 tabloids and broadsheets were united in the assumption that the Browns should be at the centre of their reporting on IVF. The only alternative story presented was a few swipes at the *Mail* about the morality of ‘chequebook journalism’, which were much more prevalent in the broadsheets than the tabloids. This demonstrates a tacit assumption across the press that IVF is about reproduction, parenting and marriage more than it is about science, politics or economics – and that readers would be most interested in the human face of IVF, namely the Browns. Human-interest stories encourage readers to engage with a story at an emotional level. In this case, this was reinforced by assumptions about the universal appeal of baby stories and dominant associations between heteronormativity and happiness (see Ahmed, 2010). In the narrative of the birth of Louise Brown, the happiness that surrounded the event obscured any ambivalence or concern that it might equally have raised and so IVF became, for the Browns and successful patients like them in the future, a technology of happiness and hope.

## The Value of Ordinariness: Money, Class and Technologically Assisted Parenthood

While Louise Brown was regularly referred to as a ‘miracle baby’,^5^ the overwhelming sense from the reportage of 1978 is of her and her parents’ ordinariness. Indeed, they could even be read as under-dogs, which would have made them all the more sympathetic to British readers. Their ordinariness was certainly one of the key messages from Lynda Lee-Potter’s coverage. In representing them as ordinary, she was making, and encouraging her readers to share, a judgement about their individual personal qualities, their moral values and their socio-economic standing – in short, about their respectability. As Beverley Skeggs (2002 [1997]) has established, respectability is a perennial marker of status in the UK and is seen as something to achieve by many working-class Britons because it is associated with those who are valued and legitimated by society. This and more recent work has also shown the importance of gendered and classed moralities of motherhood in working-class women’s ability to achieve respectability (Jensen, 2010; Skeggs, 2005; Tyler, 2008) and the importance of emotions in maintaining class divisions (Lawler, 2005; Tyler, 2008). The value of respectability draws attention to the knotty relationship between class, morality and parenting that is vital to understanding the way in which the British media represented Lesley and John in the birth of Louise Brown narrative in 1978.

Although the late 1960s and early 1970s were experienced as relatively affluent, the country suffered a number of economic problems during the mid-1970s, culminating in a financial bailout from the IMF in 1976. While almost half of the country’s population defined themselves as working class at this time (Sandbrook, 2012: 27), historian Selina Todd (2014: 314) writes that, ‘By the end of the 1970s, the economic and political power of the working class was rapidly declining.’ At the end of the 1970s, the UK was becoming a more aspirational and socially conservative society that promoted individualism alongside traditional ‘family values’.

If journalists had wished to portray the Browns negatively, they might have drawn attention to John Brown’s ‘easy’ fertility by focusing on the fact that he already had two daughters. Instead, although they did not hide his previous marriage or children, they highlighted the sadness that infertility, a ‘middle-class’ condition, had brought Lesley. Birth is not only a question of reproduction or kinship in British society, but is also intimately connected to social status and the class system. In much of the media coverage that focused on the Browns’ financial situation, which had been recently changed by first a small win on the football pools and then the syndication deal with Associated Newspapers, an implicit association emerged between the Browns’ infertility, their success at IVF and their social mobility.

The Browns often struggled to make ends meet in the early years of their marriage, and even spent their first (chaste) night together in a disused train carriage in a railway siding after Lesley had run away from home to be with John (Brown, 2015a). However, by 1978 they had secure jobs – Lesley in a cheese factory and John as a lorry driver for British Rail – and had bought their council house in Bristol. This was very unusual, as fewer than 10% of council tenants had bought their homes before Thatcher’s government championed the right-to-buy (Sandbrook, 2012: 695). The *Sun* explained how they had been able to afford private medical treatment with Patrick Steptoe:^6^

[John], aged 38, added, ‘All we want is to be a normal family. Having our own baby is our dearest wish.’

‘I have worked hard to get everything right for my wife and child.’

He went on: ‘I went working on the taxis until four o’clock in the morning to pay for my wife’s operations.’

‘I have scrimped and saved to get things together for this baby and I am going to do anything I do by myself.’ (Garside, 1978: 6)

Through accounts such as these, journalists presented the Browns, often in their own words, as a respectable, self-reliant couple who had overcome the misfortune of infertility and poverty through hard work and a bit of luck. For the Browns, ordinariness was something worth striving for. This is unsurprising given the deprived childhoods that they had both had and the financial hardships they faced in the early years of their relationship. It is also understandable given the way the media scrutiny affected their once quiet and private lives. As Lesley said, ‘Having a miracle was a lot to live up to. It felt as if the whole world expected me to be a perfect mother’ (Brown et al., 1979: 180).

Self-reliance, respectability and ordinariness were also important in the Browns’ ideas about parenting, reported in another article by Lynda Lee-Potter under the subhead, ‘HUMBLE’. They demonstrate a rather different take on the ethics of IVF and parenting from that which has become familiar from the subsequent public debates about ART:

The only concrete decision the Browns have made about Louise’s future is that it’s going to be simple.

[…] ‘There will be no frills’, says John. ‘If she’s got ability then she can go to college but she’ll get no preferences, no easy money. She’ll work for what she gets. She’ll earn every penny she spends.’

‘She’s going to be an ordinary girl brought up in an ordinary house as an ordinary person. If we treated her any differently from Sharon [her half-sister], it would be wrong. When Sharon was at school and she wanted a bit of spending money she got a Saturday job in a greengrocery.’

‘If children are pampered all it brings is sadness.’

[…] ‘We’ll get a new bed, and a new carpet for the passage but we won’t change because if we do we’ll change for the worse.’ (Lee-Potter, 1978b: 16–17)

As well as his own strong sense of the immorality of spoiling children, John was no doubt aware of how he and Lesley might be judged for the deal that they had made with Associated Newspapers. Steptoe and Edwards encouraged the Browns to sign the contract in order to control the intrusion of the press, which intensified as Lesley’s pregnancy progressed. Some rival newspapers reported they received £325,000, which they always denied (Brown et al., 1979: 161). Louise Brown (2015a: 37–41) has recently revealed that the family received an initial total of £60,000 (roughly equivalent to £240,000 today), followed by payments of £500 per interview with the *Mail* for her parents within the first year of her birth. Steptoe, who was upper-middle class, judged the amount they were paid by Associated Newspapers as ‘modest’ (Edwards and Steptoe, 1980: 190), but John was overwhelmed by the papers’ largesse (Brown et al., 1979: 160). While John was adamant that Louise would not be ‘pampered’, Steptoe had assumed that, as well as protecting Lesley from the press intrusion, the Browns had made the deal to ‘secure a financial future for their child’ (Edwards and Steptoe, 1980: 190; Lewis, 1978: 1). This points to John Brown and Patrick Steptoe’s rather different expectations of how money should assist parenthood and their contrasting ideas about women earning their own money.

In 1977, Jack Rosenthal’s BBC1 *Play for Today* about Viv Nicholson, *Spend, Spend, Spend* depicted a real-life couple from a similar background to the Browns whose lives were changed by a huge win on the pools. The contemporary news media reviled Nicholson for her ambition to spend all the money she and her husband had won on luxuries. For many newspaper readers in 1978, the Nicholsons’ story would have been familiar and the contrast with the Browns would have been clear: unlike the apparently feckless and ostentatious Nicholsons, the Browns spent their modest pools win on becoming a ‘normal family’ – via private healthcare, a clear marker of social mobility in a country with high-quality free healthcare for all – and they were determined to instil such thrift and self-reliance in their daughter, too.

In their depictions of the Browns, British journalists created a template of an ordinary, deserving couple who prioritised family over everything and who were prepared to do extraordinary things in order to achieve a ‘normal’ family. As Lee-Potter (1978a: 16) testified: ‘At the centre of this sophisticated furore is a working-class couple who describe themselves as ordinary but whose capacity for sacrifice, application, commitment and devotion to each other transcends anything that is commonplace.’ What journalists like Lee-Potter left implicit was that their success at IVF, and the deal with Associated Newspapers, allowed the Browns considerable social mobility – though they were determined not to let it change them for the worse.

## Discussion: The Birth of an Origin Story

Michael Mulkay (1997: 74–75) has described a dominant narrative in media stories surrounding the British embryo research debate that arose in the 1980s as one in which joy is created and suffering ended by the arrival of a ‘perfect’ baby. The baby symbolises hope for the parents, for society, which can be benignly assisted by science, and for the future. Mulkay notes that many of the tropes used in these debates drew on narratives used by infertile people, but alternative narratives were available. Had the press been less inclined to take such a straightforwardly celebratory approach in 1978, they might have approached the first live birth following IVF as a science story, focusing largely on the techniques involved and potential ‘applications’ rather than on the happiness it had brought to one particular couple. Even within the birth of Louise Brown narrative, there was room for a more complex story to emerge. For example, more judgemental journalists might have made hay over the fact that John already had two children (one of whom he had given up for adoption), that he had remained married to someone else well into his relationship with Lesley or that he had had an affair early in their relationship (Brown et al., 1979: 52). If they had taken a different approach, the *Daily Mail*, which has a record of disapproving of abortion, might have criticised the fact that Lesley, like other patients of Steptoe’s, had signed a contract agreeing to terminate her pregnancy if any abnormalities were detected in the foetus (*TV Eye*, 1978). Instead, tabloid and broadsheet journalists depicted the Browns as ordinary, humanising them by paying close attention to their emotions, normalising them through describing their needs and valorising their moral values, which were in tune with the conservatism of the time. The Browns’ ordinariness provided a powerful riposte to those who would criticise IVF as ‘playing god’.

The year 1978 was a troubled year: alongside the economic problems, industrial disputes and political quarrels, the UK and Ireland were in the midst of The Troubles, the ‘Yorkshire Ripper’ Peter Sutcliffe was ravaging northern England, there was widespread tension over immigration and concerns about changing social and moral norms. As the country was being pulled in many different directions, the news media united around the birth of blonde, blue-eyed Louise Brown. As well as being the happy ending for one ordinary working-class couple, this was a world-first scientific breakthrough at a time when the country was in the economic and political doldrums (see Dow, 2017). Rather than opening a Pandora’s box of unintended consequences, in 1978 IVF was represented as a way of helping married heterosexual couples have ‘ordinary’ families. It emerged as an apparently politically neutral technology and in the vast majority of reporting there was little hint of what ethical questions it might prompt in terms of embryological research or its potential to radically alter notions of family and parenthood. This may be one reason why Margaret Thatcher supported IVF behind the scenes once she became prime minister (Mulkay, 1997: 42). The story of an ordinary, self-reliant couple like the Browns using science to achieve their aspiration for a ‘normal’ family life would have chimed with Thatcher’s vision of society, which was embodied in the editorial stance of the *Daily Mail* (Addison, 2017: 189).

In the 21st century, we are familiar with popular, and pejorative, images of ‘career women’ freezing their eggs in order to delay childbirth and affluent couples procuring the services of foreign fertility clinics. The contrast with the Browns, a working-class couple who had never been abroad in their lives and who were only able to try IVF because of a fortuitously timed win on the pools, is striking. Looking back from the 21st century, it is somewhat surprising that a highly conservative newspaper like the *Daily Mail* would have framed Lesley and John Brown as ‘ordinary’ given their backgrounds or that they would have embraced IVF as a morally unproblematic technology. The fact that the *Mail* invested in the exclusive rights to the Browns’ story suggests that they believed that, not only was this an important story, but that it was one that should primarily be told from the perspective of the parents’ quest for a child of their own. This implies an assumption, which was shared by other newspapers, that this was first and foremost a human-interest story about a couple finally having their miraculous yet ordinary baby.

The birth of Louise Brown quickly became the dominant origin story of IVF, despite the many steps that had been taken to get to her birth in 1978, and the many developments that came after it. This had important consequences for how the public debates leading to the eventual regulation of ARTs in the 1980s were presented, but it also meant that any position of nuance or ambivalence, not to mention feminist critique, was squeezed out of the media frame long before those parliamentary debates began. The fact that British newspaper journalists focused on the Brown family in their coverage of the advent of IVF in 1978 suggests there was a willingness to accept this technology in a context of concern about decaying family values. Focusing on Lesley and John Brown’s appropriate emotions, modest personalities and clear moral values was a highly effective – and affective – way of telling the story of IVF because it made this technology familiar, domestic and ordinary. In this dominant narrative of the birth of Louise Brown in the British press, IVF was not a dystopian technology, an attempt to wrest control over reproduction from women, a platform for the commodification of cellular life or a means for queering parenthood, but simply a way of making babies.

## Notes

1. https://mosaicscience.com/story/growing-worlds-first-test-tube-baby/index.html. See also Brown (2015a, 2015b).

2. See Charlotte Faircloth and Zeynep Gurtin’s recent article (2017) in this journal that argues for greater dialogue between scholars of assisted reproduction and of parenting.

3. As I discuss in another article (Dow, 2017), Edwards, the research scientist, and Steptoe, the consultant obstetrician and gynaecologist, are typically referred to as the ‘pioneers’ of IVF in media and academic accounts. Edwards’ research assistant, Jean Purdy, who died young in 1985, is usually overlooked, though many of the clinicians and researchers associated with Steptoe and Edwards describe her as indispensable both in terms of her clinical expertise and the support she provided to Edwards and Steptoe (Johnson and Elder, 2015).

4. Steptoe and Edwards had attracted fear and criticism when they had announced in *Nature* that they had successfully fertilised an egg in vitro a decade earlier and this re-emerged in the 1980s in the context of the government’s Committee of Inquiry into Human Fertilisation and Embryology. During 1978, the press quite frequently reported that IVF had provoked controversy, but it rarely specified where such criticism had come from or aired any substantial objections, which leaves the impression that journalists were aware of what the existing ethical objections to IVF might be but were not particularly interested in them.

5. ‘Miracle baby’ is often a shorthand for babies born through ART now, but in the 1970s, it seems to have had a much wider usage, as there were other articles I came across while collecting data that used this term to describe a range of ‘miraculous’ births or pregnancies that did not involve IVF.

6. There is some confusion over whether the Browns continued to pay for their treatment once Lesley’s pregnancy was established, but they certainly paid for their initial consultations, as well as bearing the expense of repeated trips between Bristol and Oldham (see Brown et al., 1979: 105).
